# Multicomponent Velocity Measurement for Linear Sprinting: Usain Bolt’s 100 m World-Record Analysis

**DOI:** 10.3390/bioengineering10111254

**Published:** 2023-10-26

**Authors:** Stanislav Štuhec, Peter Planjšek, Milan Čoh, Krzysztof Mackala

**Affiliations:** 1 Faculty of Sport, University of Ljubljana, Gortanova Ul. 22, 1000 Ljubljana, Slovenia; stanko.stuhec@fsp.uni-lj.si (S.Š.); milan.coh@fsp.uni-lj.si (M.Č.); 2 Ljubljana School of Business, Management and Informatics, Tržaška Cesta 42, 1000 Ljubljana, Slovenia; peter.planjsek@sportis.si; 3 Faculty of Physical Education and Sport, Wroclaw University of Health and Sport Science, Ul. Paderewskiego 35, 51-612 Wrocław, Poland

**Keywords:** sprinting, maximum velocity, laser measurement, kinematic analysis, functional asymmetry

## Abstract

The purpose of this report is to provide additional analysis and commentary on the men’s 100 m world record of 9.58 s, set by Usain Bolt in the 2009 Berlin World Championships in Athletics. In addition, the entire race underwent a unique kinematic analysis, particularly emphasizing the maximum running velocity and its related factors. It was possible due the application of the new Stuhec software. The data were provided by LAVEG’S advanced laser measurement technology based on positional data with a high spatiotemporal resolution. The maximum velocity phase is the most critical determinant of the final race time. Bolt completed two phases in this world-record 100 m sprint: acceleration and top velocity. The borderline between these phases reached the highest velocity of 12.32 m/s on a 52 m run. He could keep the maximum velocity in five 10 m sections (50–100 m). The occurrence of functional asymmetry—the difference in step length between the left and right legs—was also noticed. Longer steps were taken with the left leg, almost over 80 m. From a practical point of view, new technologies (e.g., software) allow coaches and athletes to analyze the kinematic parameters of sprinting even more precisely and in detail. They must take into account precise changes in the course of maximum speed and the parameters determining it which are step length and frequency. Based on such an analysis, it is possible to modify the training process aimed at increasing the potential, both maximum speed and the supporting factors of strength and power. This must be conditioned by the appropriate selection of training measures shaping the abovementioned motor skills and parameters describing the optimal sprinting technique.

## 1. Introduction

The athlete Usain Bolt does not need an introduction. He is recognized all over the world for his sporting achievements. He is the winner of eight Olympic gold medals and is the world record holder in the 100 m (9.58 s), 200 m (19.19 s), and 4 × 100 m relay (36.84 s). Bolt’s 100 m world record was one of the most significant sprinting achievements, mainly due to the significant improvement in time (by 0.11 s) recorded in this run [1]. This feat has been the subject of numerous scientific studies, mainly biomechanical ones.

In the recent decade, many publications have been devoted to the possibility of an accurate analysis of Bolt’s world record [2,3,4]. However, some studies attempted to explain Bolt’s performance using spatiotemporal parameters [1,5,6]. This analysis was performed using of advanced biomechanical motion analysis software. Such diagnostics made it possible to monitor changes in the values of the basic kinematic parameters of the sprint, mainly running velocity, step length, and step frequency. The Faculty of Sport, University of Ljubljana, recently developed an entirely new approach to the kinematic analysis of linear sprint running [7]. The entire analysis is based on a laser measurement of the athlete’s displacement from the measuring device. Thanks to completely new algorithms and the already mentioned fundamental variables of running, it is also possible to calculate single-leg stance time, flight time, and step time within individual steps.

The laser distance measurement (LDM) device [8,9,10], along with the new kinematic analysis software, is a new chapter in developing velocity training control methods. This technology was used in the world’s most critical track and field competition—the 2009 World Championships in Berlin [1]. Bolt’s kinematic data during a competition are rare, and a more detailed study of Bolt’s whole-body kinematics can help confirm some of the numerous theories of his success in the sprint race. Therefore, the performance analysis with the new software is one of the most interesting procedures that can be used for biomechanical sprint analysis. We understand that a comparison by different methods of Usain Bolt’s 100 m world record has not yet been reported. Breaking down the results into the main factors determining the maximum linear velocity may provide insights that were impossible to indicate. This method is unique because it is noncontact (we did not put any measuring devices and accessories on the athlete) and can be used in competitions.

The software developed by Stuhec et al. [7] contains three phases to determine the timing of the final 100 m race [11]. The terms relating to acceleration and maintenance describe a sprinter’s performance during these race phases [12,13,14]. That is the initial acceleration right after leaving the starting block when a sprinter aims to reach maximum velocity and an upright position as quickly as possible (driving phase). After that, the sprinter finally tries to maintain top velocity for as long as possible (the maintenance phase), avoiding a drop in velocity due to increasing fatigue in the final stage of the sprint. There is, however, a human limit to achieving that maximum velocity and, in particular, to keep it for as long as possible [15,16,17].

These three phases can be used to gain insight into crucial moments in a race and, most importantly, affect a sprinter’s performance. Therefore, this new software can provide sprinters and coaches with a comprehensive understanding of their performance in a 100 m run. It is enough to measure the instantaneous velocity during the entire run. Then, the software is started to obtain additional factors determining the course of the variability in the maximum velocity over the whole distance divided into smaller sections. This experiment used the new software to analyze the variability in Usain Bolt’s 100 m world record maximum velocity in detail. Another goal was to determine which of the three running phases is considered the most critical determinant of the final race time. It has been hypothesized that at crucial points in the race, both in the complete acceleration phase and the maximum velocity hold phase, significant differences can be identified between the two phases, explaining the possibilities for an improvement of 9.58 s.

## 2. Material and Methodology

### 2.1. Subject

The time measurement in these studies was made for the world record in the 100 m race [1]. The world record belongs to Usain Bolt (23 years old, 1.96 m body height, 94 kg body mass, and 23.4 BMI (kg/m^2^), representing Jamaica [6]. He achieved 9.58 s (wind: + 0.9 m/s) at the World Track and Field Championship in Berlin on 16 September 2009. This result earned him a gold medal. Furthermore, because a human was involved in the study before the kinematic analysis was performed, the Human Ethics Committee of the University of Ljubljana approved the experiment.

### 2.2. Laser Measurement of the Bolt’s 100 m World Record

The available IAAF material obtained the data for reanalyzing Usain Bolt’s world record by applying the new straight-line kinematic analysis software. The velocity measurement was made during the final 100 m race during the World Athletics Championships in Berlin in 2009. The procedure is based on the infrared laser measurement of the distance to the athlete. A detailed description of the performed measurement was described by Graubner and Nixdorf [1] and is presented below. Three laser measurement systems (LAVEG Sport and LDM 300, Jenoptik, Jena, Germany) were used during the men’s 100 m final. The system was placed on a special tripod behind the starting line so that the light beam determines the distance to an object at any point in time, i.e., the sprinters, during the 100 m race. During the measurement, a precise lens and a crosshair focus on a point in the athlete’s lumbar region follow that point during the complete run, right through the finish. The measurement was conducted at 100 Hz (LEM 300). Therefore, from the time curve, the split and interval times can be calculated at a more satisfactory resolution (10 m intervals), as well as the mean interval and momentary velocities.

### 2.3. Software for Kinematic Analysis

The software applied by Graubner and Nixdorf [1] allowed for the split and interval times evident in the distance–time curve to be calculated at a more satisfactory resolution (10 m intervals) as well as the mean interval velocities and the momentary velocities. A team of scientists from the Faculty of Sport, University of Ljubljana, developed an even more advanced software for kinematic analysis of movement structure. The detailed description of the software’s operation was published in the authors’ previous article on the application of the laser linear distance–speed–acceleration measurement system and sport kinematic analysis software (detailed description of the software’s operation was published in the previous article by Štuhec et al., 2022) [7]. Thanks to entirely new algorithms and fundamental sprinting velocity variables mentioned above, the software allows the calculation of additional parameters. This applies to the basic kinematic parameters of a sprint step, such as step’s ground contact time, single-leg stance time, flight time, step time, stride time, and frequency. The complexity and possibilities of data acquisition by the software forced the authors to divide the analysis of Bolt’s run into two independent parts. In the abovementioned article, only the course of speed variability and the factors determining it, such as step length and frequency, were comprehensively analyzed. In turn, the distribution of forces in each step, as well as the times of the support phase and the flight phase and the time of performing a single step will be presented in the second manuscript.

The software also allows us to analyze selected run sections’ acceleration and braking phases. It eliminates unnecessary disturbances in the mechanics of the running step, providing greater mechanical efficiency of running at maximum velocity. This allows for a deeper insight into the kinematics of the sprint run, and above all, it allows us to combine different technologies into one software package. The obtained data will provide insights into multicomponent analysis that can significantly improve the training regimens of world-class sprinters.

### 2.4. Statistical Analysis

In the analysis, descriptive statistics were applied. It included the calculation of mean, SD, and V (variation). All data were analyzed using the statistical package for Windows Statistical Package for Social Science (v. 11.0, Chicago, IL, USA).

## 3. Results

Table 1 shows the basic kinematics of Bolt’s record-setting 100 m sprint. Bolt completed the run in 9.58 s, a new world record. The average running velocity was 10.44 m/s. In the race, Bolt took 41 steps, 3.91 less than the other finalists. The average step length, calculated by the actual length of each step, was 2.449 m. Bolt reached the longest step, 2.872 m, between 60 and 70 m of the run. The step length is inextricably linked to its frequency. The average step frequency differed from the actual frequency by 0.033 Hz. Comparing Bolt to the other seven finalists, there is a difference in average step length of 0.21 m in his favor. However, there is little difference in step frequency. The finalists showed a higher frequency, with a difference of 0.058 Hz. This shows how well Bolt handles these two kinematic variables, resulting in a top velocity of 12.32 m/s reached on a 52.51 m sprint.

**Figure 1:**
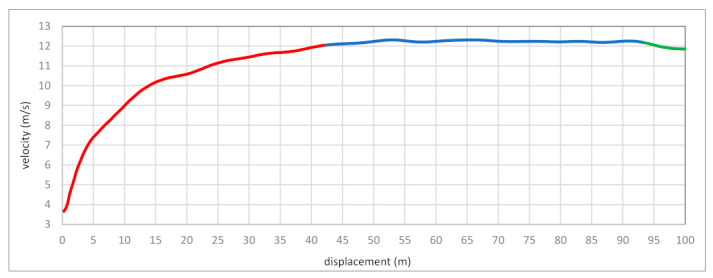
The 100 m sprint can be divided into three main phases: acceleration, maximum velocity, and deceleration. Legend: red line—acceleration, blue line—maximal velocity (2% tolerance from maximum velocity), and green line—deceleration.

Figure 1 shows the graphical course of the variation in the maximum velocity of the run by Usain Bolt divided into three phases: accelerating, reaching top velocity, and reducing this velocity. Diagram 1 shows the change in velocity versus time divided into 0.5 s intervals (above), and the caption below describes the shift concerning the traveled space—every 5 m. There are apparent similarities between the two charts mentioned above.

On the other hand, Table 2 shows the numerical course of changes in the maximum velocity in time and space. The acceleration phase lasted the longest, totaling 48.79% of 9.58 s covering 100 m. The top-velocity phase was slightly smaller, but it showed the longest distance of its run, as much as 51.79 m. The lowest values of 5.5 m showed the phases of the decrease in maximum velocity.

**Figure 2:**
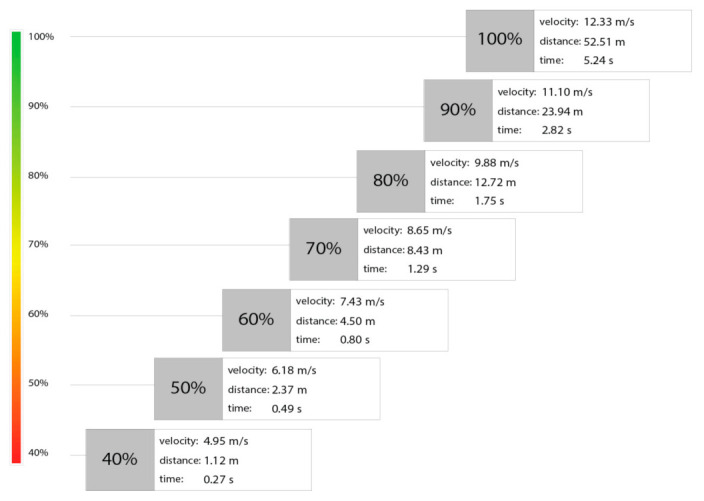
Percentage distribution of maximum running velocity.

Figure 2 presents changes in the maximum running velocity divided into percentage values. Bolt reached maximum velocity at 52.51 m, and it was 12.33 m/s. This velocity was achieved in 5.24 s. For example, compared to 60% of the maximum value, this velocity was lower by 4.90 m/s and occurred in 4.5 m, i.e., in the initial acceleration zone (0.80 s).

Table 3 presents the acceleration and maximum velocity course divided into 10 m sections. This applies to both time and space. The highest acceleration value was at the first 10 m and was higher than the second 10 m by as much as 2 m/s^2^. The decrease in the acceleration value between individual 10 m sections lasted up to 50 m, where it was 0 m/s^2^. From that moment, there is a lack of acceleration, and the last 30 m shows negative values—deceleration. Even greater differences can be seen when we analyzed the acceleration values obtained at the end of each 10 m section. In turn, the velocity at the end of the section, i.e., the real one, was compared with the average velocity for a given section. Times were compared on individual sections that were accumulated. It is noticeable that for up to 60 m of the 100 m sprint, the velocity at the end of each 10 m section was higher than the average for this section; e.g., for 60 m, the velocity at the end was 0.22 m/s higher than the average. The trend changed, starting with a 70 m run, where the following three 10 m sections showed a higher average velocity. The last 10 m of the run had the same maximum velocity of 11.11 m/s. Therefore, we noticed that after 50 m of the run, Bolt entered the maximum velocity phase.

**Figure 3:**
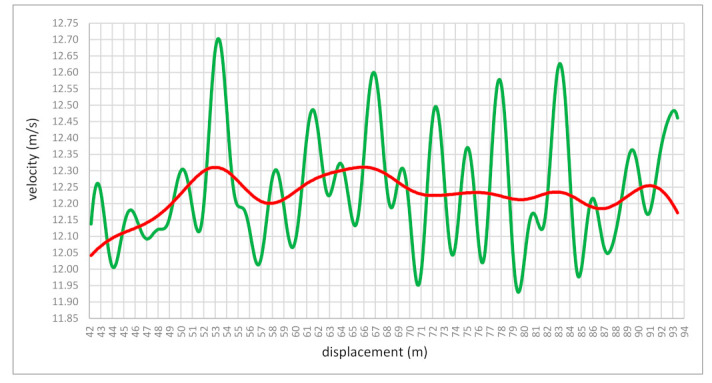
The course of the variability in the maximum velocity in the phase of top running velocity. Plotting a velocity–displacement curve. Green line—raw velocity data and red line—smoothed velocity data.

Figure 3 shows the course of the velocity variability in the phase of the maximum velocity of Bolt’s sprint run, obtained between 40 and 84 m. The duration of the total phase (after covering 40.01 m) was 3.38 s, where the runner covered a distance of 44.05 m at a velocity above 11.68 m/s. A detailed analysis of the maximum running velocity phase shows a division into a longer subphase of developing the highest velocity lasting 2.32 s (distance covered 26.73 m) and a shorter subphase of reducing velocity lasting 1.51 s.

**Figure 4:**
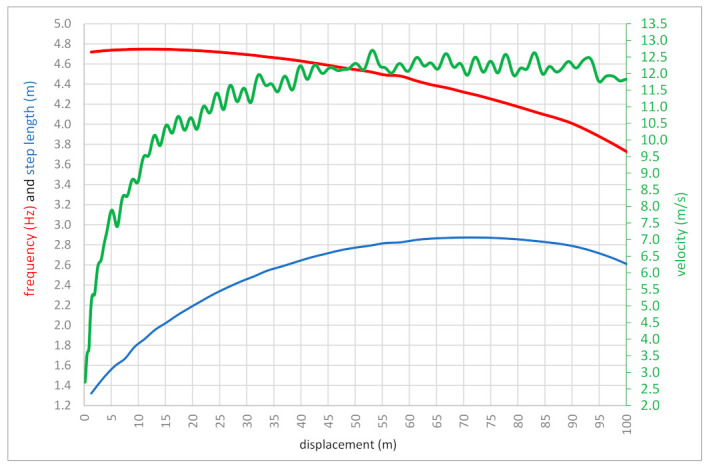
Dynamics of frequency change, step length, and velocity depending on displacement. Red line—step frequency, green line—velocity, and blue line—step length.

Table 4 and Figure 4 show the relationship between the length and frequency of steps during Bolt’s record run. The classic relationship between these two kinematic parameters can be seen as the length of the step decreases. In this run, Bolt took 41 complete steps with an average length of 2.44 cm and a frequency of 4.47 Hz. The highest frequency of steps was at the beginning of the run in the initial acceleration phase. The longest step of 2.86 m was performed on a 69.82 m run at a frequency of 4.32 Hz. The longest steps were executed in the maximum velocity phase between 56 and 86 m of the 100 m sprint with an average length of 2.84 cm.

## 4. Discussion

This report provides additional analysis and commentary on the men’s 100 m world record of 9.58 s, set by Usain Bolt. In addition, the entire race underwent a unique kinematic analysis, with particular emphasis on the maximum running velocity. This was possible due to the application of new software. The data were provided by LAVEG’S advanced laser measurement technology based on positional data with high spatiotemporal resolution. Because Bolt’s velocity was measured with the laser only once, this must be used in the future as a reference for other sprints by him and world-class sprinters.

The question is how the raw and precisely processed data of one athlete—Usain Bolt—can be used to improve sprint performance globally—for all sprinters. This seems unreasonable. The problem is the inability to compare Bolt’s data with the data of other sprinters. This results from the failure to maintain measurement reliability due to applying different methodologies for obtaining and processing raw data [18,19]. Are these methodologies so different that comparing the data and conducting a detailed analysis is impossible? The latest publication by Healy et al. [20] and earlier by Mackala and Mero [6] and Graubner and Nixdorf [1] can solve the problem. Data acquisition by Healy et al., 2022 [20] combines video measurements (sampling rates: 50–250 Hz) and laser measurements (sampling rates: 50–100 Hz). Size at such a high frequency gives comparable results [19]. Healy’s [20] data compare actual (measured) kinematic and modeled variables. The modeled variables have excellent accuracy and strict agreement with the raw measurements (the mean deviation range was 0.2%, and the ICC range was 0.935 to 0.999). Additionally, Healy et al. [20] divided the sprinters into tertiles, based on their 100 m time, with the first and third tertiles being the faster and slower groups, respectively. Therefore, to better understand the analysis of our problem, Bolt’s data can be compared to the first tertile, which is faster sprinters (100 m time 9.91 ± 0.10 s and range 9.58–10.02 s, i.e., including Bolt’s results). It can therefore be assumed that the raw and modeled results represent almost identical values that can be compared with those obtained in the Bolt analysis.

Bolt’s 100 m world-record run was divided into three main phases: a block start and an acceleration phase, which is sometimes divided into an initial acceleration phase and the main acceleration phase, a top-velocity phase, and the last phase of slowdown, where a slight decrease in velocity can be noticed. A similar division appeared in numerous publications describing changes in the course of the maximum velocity in the 100 m run [20,21,22,23,24]. Bolt’s route of velocity changes was plotted by a velocity–time curve and a velocity–distance curve (Figure 1). These two charts illustrate his all-around performance in all three sections of the race. The unique thing is that the course of the variability in maximum velocity shows a similar graph for both plots. This means Bolt matches his performance in all three phases of his record run. We can assume that compared to the best sprinters, even those from the 1980s and 1990s (e.g., Carl Lewis and Donovan Bailey), Bolt is distinguished by velocity in the acceleration phase. Graubner and Nixdorf [1] confirmed that he is ahead of his competitors in maximum velocity and sprint endurance phases, expressed by higher values of develop velocity. It could be argued that such a course of the velocity curve sets new trends. It was close to Healy et al.’s [20] work but presented a much larger population of world-class sprinters with averaged values of the analyzed kinematic parameters.

Additionally, Bolt’s 100 m performance analysis differs considerably from his previous analyses and those of world-class sprinters. It concerns the times for the measured 10 m intervals. He recorded five sections of 10 m (between 50 and 90 m) where the average velocity value in the 10 m section reached over 12 m/s, ranging from 12.10 to 12.32 m/s (Figure 3). On the other hand, the analysis performed by Graubner and Nixdorf [1] showed only four such sectional measurements between 40 and 80 m. In turn, the previous work of Mackala and Mero [6] also presented five sections over 12 m/s, with the highest value of 12.34 m/s between 70 and 80 m. The time of the seven 10 m sections was the beloved 0.90 s (Figure 3). The same was noticed by Mackala and Mero [6]. As can be seen, all measurements taken by those researchers were within a tiny margin of error. His average velocity was 10.438 m/s, which does not give the complete picture of velocity ability because each sprinter’s top velocity is much higher than the average velocity. In the case of Bolt, our analysis showed that his maximum velocity was 12.32 m/s. Figure 4 shows how the velocity values changed in the phase of maximum running velocity. Generally, he had a length of about 50 m, where the velocity of entry into this zone was 12.05 m/s and the velocity of exit from the area was 12.17 m/s. It means that for about 50 m, the velocity practically stabilized at the same level. The difference was only 0.12 m/s. The question is, is it possible to maintain maximum velocity over such a long distance? Practice shows that such high-velocity values can be carried over three 10 m sections. With Bolt, it was five 10 m episodes. What was this caused by? The explanation of this phenomenon should go in a few directions.

The main direction is that Bolt is fast because he can run at maximum velocity for longer. Bolt’s advantage over other world-class sprinters is that he quickly reaches a very high maximum velocity of 40–50 m and maintains it as long as possible—another 50 m. It means that the acceleration phase is a crucial element of the 100 m race. To understand this relationship, we must carefully trace the percentage velocity distribution of his record-breaking 100 m race. According to Figure 2, Bolt’s initial acceleration is characterized by a rapid increase in velocity up to 11.10 m/s (about 24 m or 2.82 s, representing 90% of the maximum rate), then turning into a phase of gradual increase in velocity. The following 10% of velocity, which closes with a maximum value of 12.32 m/s, is achieved in the next 25 m (52.51 m run distance). This distance ends the pick-up phase. A subsequent maximum velocity phase represents a more constant velocity with tiny fluctuations in its maximum value (100%) over the next 50 m. Compared to Brüggemann et al. [25], Ae et al. [26], and Haely et al. [20], all analyzed sprinters reached their highest velocity between 50 m and 80 m. The last 20 m was the deceleration phase, represented by a gradual decrease in velocity. However, the work of other authors [6,13,17,23] clearly shows that faster sprinters usually reach maximum velocity later in the race, 65–70 m and they can hold it for about 30 m. Therefore, shifting the limit of reaching the maximum velocity to 65–70 m for world-class sprinters and the absence of the velocity reduction phase for Bolt significantly affect the results achieved in the 100 m sprint.

The second direction is maintaining the correct relationship between the length and frequency of steps in the maximum velocity phase. Gejer et al. [27], Donati [28], Majumdar and Rogers [12], Debaere, et al. [29], Ballreich [30], and Mackala and Mero [6] analyzed these two parameters in detail. They showed that only two 10 m sections, between 60 and 70 m and 80 and 90 m, showed a constant velocity, indicating that the same proportions (values) between the length and frequency of steps were maintained in both cases. This was not confirmed by our recent analysis using the new software. However, an exciting relationship occurred in the first 30 m, broken into 10 m sections. The stride frequency was almost at the same level, and the difference was only 0.03 Hz when a maximum of 47 cm extended the stride length (Table 2). The reverse relationship was in the phase of maximum velocity. In four 10 m sections, the stride almost stabilized at the same length (differences of 5 cm) with a significant frequency fluctuation between 0.10 and 0.41 Hz. Therefore, the possibility of a negative or positive interaction between the two discussed kinematic parameters is limited [6,20,28]. In Bolt’s run, a functional asymmetry is noticeable, i.e., a difference in the length of the step with the left leg push compared to the right leg push. This is because more extended efforts are made with the left leg taking off. Another regularity can also be noticed here: in up to 80 m of the run, each subsequent step, regardless of which leg is taken off, shows an upward trend, i.e., is longer. This confirms the observations of Nummela et al. [31], who reported that 90% of a runner’s velocity is attributed to stride length, and anything after that increases through frequency. The last 20 m decrease in stride length also maintains the previous trend. This observation is made possible by determining the actual length of each step. This cannot be noticed when analyzing the average size of steps, e.g., from a 10 m section.

The abovementioned observation coincides with the conclusions of Udofa et al. [32] 2017, who assessed Bolt’s pattern of ground force application and measured the impulse for each leg. He determined that how Bolt achieves his instincts seems to vary from leg to leg. It means he had a different value, which allows us to say that the applied power in each step gives its extra length. Although using two other measurements (stride length and impulse) and Udof’s [32] observation from another run (IAAF meeting in Monaco), the conclusions regarding the change in stride length are the same. Therefore, like Udof’s [32] observation, our findings also raise the immediate scientific question of whether a lack of symmetry represents a personal mechanical optimization that makes Bolt the fastest sprinter, better than the rest of the world. There may also be other factors causing asymmetry, which have not yet been fully defined, that relate to the reaction of the nervous system to fatigue, a decrease in impulse strength, or the exact determination of the anatomical value of the difference in the asymmetry between the left and right legs. Does this asymmetry negatively affect the achieved result; i.e., if this asymmetry were eliminated, would Bolt run even faster?

In the end, one should consider the limitations of this analysis. As previously mentioned, can the analysis of one athlete, even the best in the world, be used as an interpretation for other sprinters to improve their performance? Perhaps the conclusions of Beneke and Taylor [3] are worth considering. They found that in the case of Bolt, a combination of a likely large percentage of fast-twitch fibers and remarkable anaerobic capacity can create a powerful and fast push-off with every step, developing the very high value of velocity and keeping it for very long time/distance.

In addition to these factors, it should also be considered that behind Bolt’s efficient movement, a different running technique separates him from his rivals. We are talking about the angular analysis of body segments based on muscular efforts directed to the active movement of legs pushing (generation of force) the body forward. In other words, he can apply more mass-specific force to the ground at the same or shorter time [14]. All these elements are essential to running fast, no matter the sprinter’s athletic level. The higher the level, the more critical they become due to the interactions they have to enter into with each other. Additionally, it is crucial to emphasize that Usain Bolt’s remarkable stride length, attributed to his unique anthropometry, stands out as a pivotal differentiating factor when compared to other finalists in the 100 m run. This combination presents an athlete who not only possesses an exceptional stride length but also maintains a high stride frequency—a rare and challenging characteristic to find across sprinters.

## 5. Conclusions

The detailed analysis shows that the maximum velocity phase is the most critical determinant of the final race time. In this phase, Bolt maintained his top velocity over five 10 m stages, clearly different from other world-class sprinters. Furthermore, there is no noticeable decrease in velocity in the last 10 m of the run, which indicates that Bolt completed two phases in this run: acceleration and maximum velocity. The borderline between these phases was achieving the highest rate of 12.32 m/s on a 52 m run.

Although the maximum velocity between the 50 and 100 m run in five 10 m sections was almost constant, the rule of keeping the same proportions (values) between the length and frequency of steps was not confirmed. The reason for this was the occurrence of functional asymmetry—the difference in step length between the left and right legs. Longer steps were taken with the left leg. In addition, a continuous increase in stride length was observed for the left and right legs up to 80 m sprinting.

From a practical point of view, new technologies (e.g., software) allow coaches and athletes to analyze the kinematic parameters of sprinting even more precisely and in detail. They must take into account precise changes in the course of maximum speed and the parameters determining it which are step length and frequency. Based on such an analysis, it is possible to modify the training process aimed at increasing the potential, both maximum speed and the supporting factors of strength and power. This must be conditioned by the appropriate selection of training measures shaping the abovementioned motor skills and parameters describing the optimal sprinting technique.

## Figures and Tables

**Table 1:** Numerical characteristics of selected kinematic parameters of Bolt’s world-record 100 m sprint compared to the rest of the finalists.

Kinematic Parameters		Usain Bolt World Championships Berlin 2009 *	Rest of Finalists World Championships Berlin 2009 **		
			$\bar{X}$	SD	V
Time [s]		9.58	9.91	0.10	1.06
Velocity [m/s]		10.44	10.09	0.11	1.01
Step frequency [Hz]		4.472	4.53	0.20	4.41
Number of steps	All	41.00	44.91	1.77	3.94
	Right leg	21	-	-	-
	Left leg	20	-	-	-
Step length [m]		2.449	2.23	0.09	3.93

* data from Stuhec’s software v. 3.0, ** data from IAAF—Berlin 2009.

**Table 2:** The numerical course of changes in the maximum velocity in time and space.

	Acceleration		Maximum Velocity		Descending Velocity	
	Phase		Phase		Phase	
duration (s):	4.44	48.79%	4.19	48.04%	0.47	5.16%
from–to (s–s):	0.00–4.44		4.44–8.63		8.63–9.10	
distance (m):	42.80	42.95%	51.28	51.47%	5.56	5.58%
from–to (m–m):	0.00–42.80		42.28–94.07		94.07–99.63	

Legend: red—acceleration, blue—maximal velocity (2% tolerance from maximum velocity), and green—deceleration.

**Table 3:** Division of Bolt’s 100 m sprint acceleration and velocity (on point and an average) into 10 m sections.

Distance (m)	Acceleration on the Point (m/s^2^)	Average Acceleration between 10 m Sections (m/s^2^)	Velocity on the Point (m/s)	Average Velocity between 10 m Sections (m/s)	Time on the Point (s)	Time between 10 m Sections (s)
10	2.600	3.900	9.12	6.80	1.46	1.46
20	0.700	1.900	10.60	10.17	2.45	0.99
30	0.600	0.975	11.46	11.12	3.35	0.90
40	0.600	0.513	11.95	11.71	4.29	0.85
50	0.350	0.412	12.25	12.10	5.03	0.83
60	−0.350	0.000	12.32	12.23	5.85	0.82
70	−0.150	0.087	12.32	12.32	6.66	0.81
80	−0.080	−0.137	12.24	12.20	7.48	0.82
90	−0.150	−0.075	12.26	12.23	8.30	0.82
100	−0.350	−0.350	12.27	11.84	9.11	0.81

**Table 4:** The relationship between the length and frequency of steps during Bolt’s record sprint. The division into 10 m sections.

Section (m)	Number of Steps	Step Length (m)	Step Frequency (Hz)	Average Step Time (s)	Real Distance (m)
10	6	1.55	4.73	0.111	9.31
20	5	2.02	4.74	0.117	19.43
30	4	2.34	4.71	0.122	28.79
40	4	2.56	4.66	0.120	39.03
50	4	2.72	4.58	0.124	49.93
60	3	2.81	4.49	0.119	58.36
70	4	2.86	4.34	0.129	69.82
80	3	2.86	4.24	0.123	78.42
90	4	2.82	4.08	0.122	89.71
100	4	2.68	3.38	0.168	100.44

## Data Availability

The data presented in this study are available on request from the corresponding author.
